# Horizons in the evolution of aging

**DOI:** 10.1186/s12915-018-0562-z

**Published:** 2018-08-20

**Authors:** Thomas Flatt, Linda Partridge

**Affiliations:** 1 Department of Biology, University of Fribourg, Chemin du Musée 10, CH-1700 Fribourg, Switzerland; 2 Max Planck Institute for Biology of Aging, Joseph-Stelzmann-Strasse 9b, D-50931 Cologne, Germany; 3 Institute for Healthy Aging and GEE, University College London, Darwin Building, Gower Street, London, WC1E6BT UK

## Abstract

Between the 1930s and 50s, evolutionary biologists developed a successful theory of why organisms age, firmly rooted in population genetic principles. By the 1980s the evolution of aging had a secure experimental basis. Since the force of selection declines with age, aging evolves due to mutation accumulation or a benefit to fitness early in life. Here we review major insights and challenges that have emerged over the last 35 years: selection does not always necessarily decline with age; higher extrinsic (i.e., environmentally caused) mortality does not always accelerate aging; conserved pathways control aging rate; senescence patterns are more diverse than previously thought; aging is not universal; trade-offs involving lifespan can be ‘broken’; aging might be ‘druggable’; and human life expectancy continues to rise but compressing late-life morbidity remains a pressing challenge.

## The evolution of aging in humans

Human life expectancy worldwide has increased dramatically. During the ~ 300,000 generations since the divergence from our most recent common ancestor with the great apes, lifespan evolved to double its previous value [1]. In the last ~ 200 years there has been a further substantial increase, on average about 2.5 years per decade, attributable to environmental changes, including improved food, water, hygiene, and living conditions, reduced impact of infectious disease with immunization and antibiotics, and improved medical care at all ages [2–5]. As a result, most people are now living long beyond the ages at which most would have been dead in the past. Natural selection has therefore not had an opportunity to maintain evolutionary fitness at older ages. Presumably as a consequence, advancing age is the major risk factor for diverse types of loss of function, and for highly prevalent chronic and killer diseases, including cancer, cardiovascular disease, and dementia [6, 7]. Consequently, healthy life expectancy has not increased as much as has overall life expectancy [8, 9], and there is a growing period of late-life morbidity before death (WHO data on life expectancy [10]).

Many modern humans inhabit a very different environment from that in which their life history evolved, with both protection from many of its dangers, such as predators, infectious diseases, and harsh physical conditions, and freedom from the need to forage extensively to avoid starvation [1]. However, the ready availability of calorie-dense food, together with the low requirement for physical exercise, are resulting in a tidal wave of metabolic disease that has a major impact at all ages, but particularly on deaths from cardiovascular disease later in life [11]. Modern humans therefore often have many features in common with laboratory model organisms, which also inhabit highly protected, calorie rich, and physically restricted environments.

**Fig. 1.**
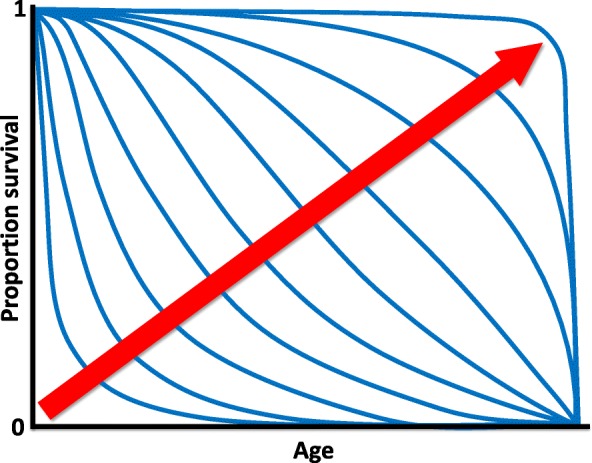
Rectangularization of survival curves. Hypothetical survival curves with different degrees of ‘rectangularization’ (as indicated by the *red arrow*): increasing ‘rectangularization’ implies a high, constant probability of survival to a very advanced age (i.e., a long ‘shoulder’ of the curve) and a marked compression of morbidity and death into a very narrow age range (i.e., an almost vertical drop in survival at the end of life)

Aging human populations have become a grand challenge to societies worldwide. The major burden of ill health is now falling on older people. Declining birth rates, together with the population bulge in some countries from the baby boomers and generally longer lives, are increasing the ratio of dependent to independent members of society, posing major economic and social problems [12]. Current demographic trends indicate that life expectancy is likely to continue to increase in all countries for which there are good data [13], and it is unclear when any limit to human lifespan will be seen (see the recent debate in [14–16]). There is hence a pressing need to find ways of keeping people healthy for longer and hence compressing and reversing the growing period of morbidity at the end of life [9, 17]. Interestingly, it has been found that late-life disability and morbidity are lower among people living beyond 100 years [18]. Morbidity can thus be restricted, at least in principle, to the very end of life (Fig. 1). In this review, we discuss what can be learned about aging by considering its evolutionary biology, and how evolutionary thinking could help inform practical measures to ameliorate the effects of human aging.

## Why does aging evolve?

Aging, or senescence, is characterized demographically by increasing mortality and decreasing reproductive success with advancing adult age [19–21]. These effects of aging, and other types of age-related loss of function, have been extensively documented under field conditions [22]. Aging manifests itself most clearly under benign environmental conditions in captivity, since in the wild individuals of many species are hard to track throughout life (but see [23]), and high rates of age-independent mortality (e.g., due to predators, pathogens, food shortage) can obscure the intrinsic tendency of adult survivorship and fecundity to decline with age [19, 21, 24]. The occurrence of aging in nature poses an evolutionary puzzle: why would such a deleterious, maladaptive process evolve [20]? This puzzle is deepened by the fact that aging is apparently neither inevitable nor universal: germ lines and several organisms do not exhibit senescent decline [21, 25–27].

**Fig. 2.**
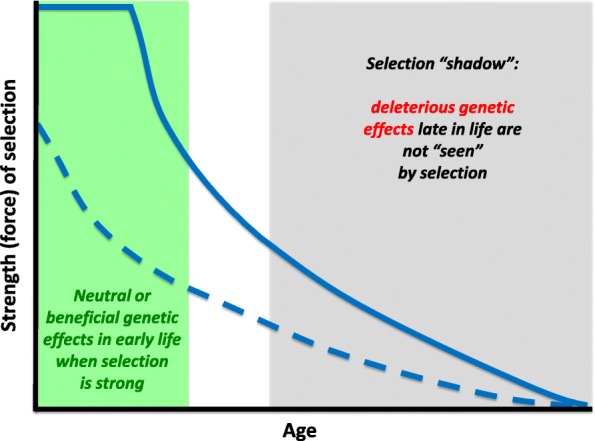
The declining force of selection. The strength (‘force’) of selection measures how strongly natural selection acts on changes in survival and/or fecundity. Often, but not always, the force of selection declines with age. If this is the case, then alleles with neutral effects on fitness early in life but with deleterious effects late in life can accumulate in a population, unchecked by selection (mutation accumulation). Similarly, alleles with positive effects on fitness components early in life can be selectively favored even if they have negative effects late in life (antagonistic pleiotropy). The late-life negative effects in the ‘selection shadow’ cannot be effectively eliminated by selection, leading to senescence. While the force acting on survival (*solid line*) only starts to decrease with age after the onset of reproduction, the strength of selection on fecundity (*dashed line*) can increase or decrease before the onset of reproduction (for details see references [36, 38])

The basic puzzle of why organisms age was addressed in a series of trail-blazing studies published between the 1930s and 1950s [19, 24, 28]: Fisher [29] and Haldane [30] were the first to realize that aging results from natural selection typically having a much larger impact on survival and reproduction early as compared to late in life, a notion further developed by Medawar [31, 32] and Williams [33]. This idea was later mathematically formalized by Hamilton in 1966 [34] (also see [25, 35, 36]) (Fig. 2). Importantly, Hamilton corrected the error of using Fisher’s so-called ‘reproductive value’ as a measure of how sensitive fitness is to age-specific changes by using the ‘intrinsic rate of increase’ (also called the ‘Malthusian parameter’) as a fitness measure [19, 34, 37, 38]. The underlying driver of the evolution of aging is that various forms of ‘extrinsic’ (i.e., environmentally caused) hazards, such as disease, predation, and accidents, largely determine the adult mortality rate and hence cause a characteristic decline with time in the numbers of surviving individuals in a cohort. Genetic variants that affect fitness at later ages will therefore encounter a weakened force of natural selection, because some of their bearers will die from extrinsic hazard, at a rate no different from non-bearers, up to the age when the variant starts to manifest its phenotypic effects and hence affect fitness. The population genetic theory of aging posits that this process leads to two non-exclusive mechanisms (Fig. 2). The first is ‘mutation accumulation’ (MA), proposed by Medawar in 1952 [32]. If the force of selection declines with age, deleterious mutations whose effects are restricted to late life can accumulate to higher frequency under mutation-selection balance, due to a progressively weakened force of natural selection. J.B.S. Haldane discussed Huntington’s disease, caused by a dominant mutation, and with an average age of onset of ~ 35 years, as an example of mutation accumulation [30]. The second mechanism is ‘antagonistic pleiotropy’ (AP), a concept proposed by Medawar [31, 32] and Williams [33]. Here, selection can favor mutations or alleles with positive effects on fitness-related traits early in life, even if these same genetic variants have negative effects late in life, because selection will act less strongly against the late-life deleterious effects if its strength declines with age. Cellular senescence, a cell cycle arrest in normally dividing cells, is a potential example of antagonistic pleiotropy. The process is important during development and wound healing, where it participates in tissue remodeling. Cellular senescence is also vital in protection against cancer, because it occurs in response to DNA damage. However, during aging senescent cells, instead of being removed by the immune system, accumulate in tissues and cause damage, by secreting inflammatory molecules, and hence are important in the etiology of many aging-related diseases [6, 39]. A physiological version of antagonistic pleiotropy, the ‘disposable soma’ (DS) hypothesis, assumes that there is a physiological (energetic) trade-off between damage repair and somatic maintenance versus reproductive investment [40–42]. The mathematical theory of MA and AP was worked out chiefly by Charlesworth [19, 37, 38, 43]. An important assumption of the theory is that aging should evolve universally whenever there is a sharp distinction between parents and their offspring (or between somatic and reproductive structures); if there is no such distinction then the theory does not apply [33, 38, 44] (also see below).

A large body of experiments, mainly in the fruit fly *Drosophila melanogaster*, but also other organisms, supports the AP and MA mechanisms (reviewed in [45–53]). In particular, trade-offs between lifespan and fecundity (or other fitness components) consistent with AP have been found in artificial selection or ‘experimental evolution’ experiments performed on outbred laboratory stocks [54–59], in analyses of mutants and transgenes [46–51, 60–64], and in studies of naturally segregating polymorphisms [47, 65–67]. MA is also well supported, mainly by quantitative genetic studies [49, 68–70] (but see [71] for a critique). In humans, data from medical genetics and genome-wide association studies (GWAS) indicate that both mechanisms might play a role in explaining late-onset diseases and trade-offs between lifespan and fitness-related traits [49, 72–80]. MA is supported by a large number of dominant mutations with late age of onset, and by a recent quantitative genetic analysis of a human historical population [73, 81]. With regard to AP, for example, mutations in BRCA1/2 cause increased risk of breast and ovarian cancer yet have positive pleiotropic effects upon fertility [80]; however, since these mutations are rare, it is somewhat difficult to see how they are consistent with AP.

A classic prediction of the evolutionary theory of aging, due to Medawar [32] and Williams [33], is that low ‘extrinsic’ (i.e., environmentally imposed) adult mortality leads to the evolution of low intrinsic adult mortality (i.e., slowed aging), while the opposite is expected under high extrinsic adult mortality. This postulate was borne out in an experimental evolution experiment where fruit flies were exposed to high versus low extrinsic adult mortality [58]. Extrinsic mortality affects senescence only if it has differential effects among different age classes in an age-structured population [37, 38, 82–84], a point that was implicit in Williams’ 1957 focus on adult (as opposed to preadult) mortality [33, 52]. Indeed, extrinsic mortality often has age-dependent effects: for example, in many large mammals, juveniles and old individuals are more susceptible to extrinsic mortality than prime-aged individuals [85]. Complications can arise if extrinsic mortality affects population growth/density, or if it interacts with organismal condition; both factors can affect the rate of aging if extrinsic mortality is age-dependent. This can lead to situations where lifespan evolves to be longer, not shorter, under high extrinsic mortality [25, 86–88]. For instance, increased extrinsic mortality can select against senescence of a physiological trait that reduces the susceptibility to this source of mortality, causing the evolution of improved somatic condition and longer life; this has been confirmed in guppies and the nematode worm *Caenorhabditis remanei* [87, 88]. Yet, what remains true is that levels of extrinsic, adult mortality are a key driver of the evolution of aging [28].

Aging therefore evolves as a non-adaptive side effect of the declining ability of selection to maintain fitness at older ages. In humans, where age-related changes are particularly well documented, aging has proved to be a complex process of functional decline and accumulation of diverse pathologies in different tissues [6, 89]. Williams predicted in 1957 [33] that aging is likely to be a genetically complex trait, and different lineages and taxa might well exhibit different proximate mechanisms of senescence. Indeed, natural variation in the rate of aging is likely influenced by many genes [90–92], since survival and reproduction between them harness the activity of much of the genome.

## Some mechanisms of aging are evolutionarily conserved

Despite Williams’ prediction that “senescence should always be a generalized deterioration, and never due largely to changes in a single system” [33], aging in laboratory animals has—initially somewhat surprisingly [93]—turned out to be highly malleable to simple genetic, environmental, and pharmacological interventions. Furthermore, similar interventions seem to ameliorate the effects of aging in distantly related organisms, suggesting evolutionary conservation of mechanisms [46, 62, 94–97]. Aging in diverse organisms has characteristic hallmarks, including genetic instability, failure of key cellular processes and components, and impairments of tissue function [6, 21]. These processes can interact within cells and tissues, through action at a distance between them, and through deterioration of the aging systemic environment [6, 98, 99]. The interventions that ameliorate the effects of aging in laboratory animals slow down or suppress at least some of these aging hallmarks.

Dietary restriction (DR) is the longest established and currently most effective means of improving health during aging and extending lifespan in the laboratory. The food intake of DR animals is experimentally reduced, while avoiding malnutrition. Various DR regimes have proved effective in diverse model and non-model invertebrates and vertebrates [100–103], as a result of either conserved mechanisms or parallel evolution. In rodents and rhesus monkeys, DR improves almost all aspects of health during aging, except wound healing and resistance to certain viruses [104–108]. Reduction in intake of specific dietary components, particularly protein, rather than of overall calories, underlies the health improvements from DR [109–115]. DR animals often gorge their daily ration in one meal, and fast until the next one, and this intermittent fasting may play a role in the health improvements [116–119]. DR has been suggested to induce evolved mechanisms for surviving food shortages in nature. Fecundity is usually reduced during DR [120], and organisms short of food might thus reallocate nutrients to somatic maintenance, and hence survive the famine to reproduce more successfully with the return of the food supply [121] (but see [122] for evidence against this hypothesis). However, these results have been obtained largely with laboratory animals, while animals in natural populations often respond to food provisioning with increases in reproduction, function, and survival [123]. In nature some degree of DR may therefore be the norm [124, 125].

**Fig. 3.**
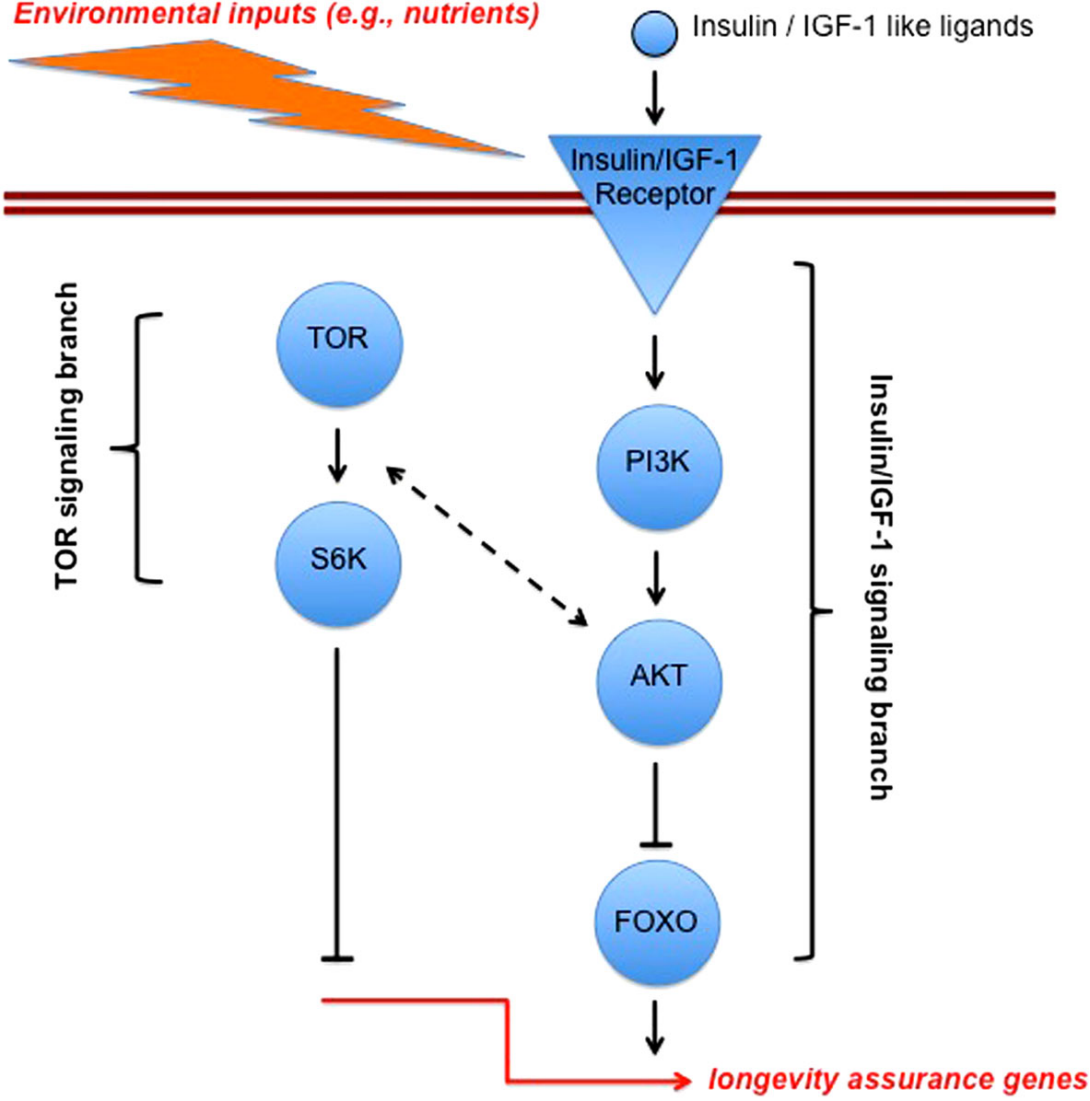
A strongly simplified representation of the evolutionarily conserved insulin/insulin-like growth factor signaling (*IIS*) and target of rapamycin (*TOR*) network which regulates lifespan in distinct organisms, from invertebrates to humans. In response to environmental inputs (e.g., nutrients) the IIS and/or TOR branches of the network become activated; reduced input (inhibition) of the signaling network leads to the activation of downstream transcription factors (such as the forkhead box O transcription factor FOXO) that regulate the expression of hundreds of target genes, many of which are involved in longevity assurance (but which also affect other life-history traits, including growth, size, and reproduction). Many of the genetically homologous components of this network have been experimentally shown to affect lifespan in *C. elegans*, *Drosophila*, and mouse; evidence from GWAS shows that genetic polymorphisms in some of these components are also associated with exceptional longevity in humans. The homologs of IIS/TOR components have different names in different species: for example, in *C. elegans*, the insulin-like receptor (INR) is called DAF-2, PI3K is called AGE-1, and FOXO is called DAF-16; in humans, the FOXO homolog associated with longevity is called FOXO3A. Note that humans, in contrast to invertebrates, not only have an insulin receptor but also an insulin-like growth factor 1 receptor (IGF-1); it is thought that the different physiological functions of the insulin receptor versus the IGF-1 receptor in humans are being subsumed by a single insulin receptor in invertebrates

Organisms sense both nutrients and their own nutritional status through multiple, parallel mechanisms. An important contributor is nutrient-sensing signaling through the highly evolutionarily conserved insulin/insulin-like growth factor signaling (IIS) and the target of rapamycin (TOR) network, which matches the costly activities of organisms, such as growth, metabolism, and reproduction, to nutrient and stress status (Fig. 3). Beginning with the isolation of the first long-lived laboratory mutants in the late 1970s and early 1980s, it was found that genetically reduced activity of IIS/TOR can increase lifespan in the nematode worm *Caenorhabditis elegans*, the fruit fly *D. melanogaster*, and the mouse *Mus musculus* [51, 61–64, 96, 97, 126–146]. These mutant animals show a prolonged healthspan and are protected against both natural aging-related decline and the pathology associated with genetic models of human age-related diseases [7]. Remarkably, genetic variants in, and altered expression of, the orthologues of the genes encoding components of this network are also associated with survival to advanced ages in humans [147–152]. The IIS/TOR network contains many potential drug targets, and rapamycin, a licensed drug that targets a protein complex in the TOR network, can extend lifespan in diverse laboratory organisms, including mice [153–157].

The IIS/TOR nutrient-sensing network might also play a significant role in aging and life history in nature. For example, multiple lines of evidence suggest that insulin-like growth factor-1 (IGF-1) signaling mediates physiological life-history variation in mammalian populations [158, 159], and in *D. melanogaster* naturally segregating polymorphisms in IIS have been linked to latitudinal gradients (clines) in life-history traits [160–163], with some natural alleles of the *insulin-like receptor* (*InR*) gene having pleiotropic effects upon lifespan, stress resistance, fecundity, and body size [164, 66]. Analysis of quantitative trait loci affecting gene expression (so-called eQTLs) in recombinant inbred fruit fly lines has also identified some IIS variants that affect transcription in response to dietary change [165]. Moreover, in honeybees and other social insects, IIS/TOR has profound physiological and developmental effects on caste development, foraging behavior, and probably longevity itself [163, 166–173].

Does the existence of such a conserved signaling network with major effects on aging contradict Williams’ assertion that senescence should not be due to a single cause or system? Evolutionary theories of aging generally do not consider the evolution of phenotypic plasticity, the ability of a single genotype to produce different phenotypes in response to changes in the environment [163]. As individuals go through life, they can encounter widely varying environmental challenges, as can different populations. Phenotypic plasticity of life history in response to varying nutrition, infection, predation, and physical stresses is therefore widespread. The consistent role of the IIS/TOR network in aging may well represent a high degree of evolutionary conservation and optimization of the mechanisms of phenotypic plasticity in life history. Moreover, while aging does result from various forms of system failure owing to limitations of defense against aging-related damage, longevity assurance is a highly regulated process of maintenance and repair. From this point of view, it might not be so surprising then that signaling pathways have evolved that can plastically and genetically match an organism’s investment into somatic maintenance, repair, survival, growth, and reproduction with the prevailing environmental conditions [163, 174].

Although the IIS/TOR network is a major regulator of life history in many taxa [46, 64, 163, 175], several mapping studies and artificial selection experiments in *D. melanogaster* have failed to identify canonical IIS/TOR genes as harboring natural variation for lifespan or other life-history traits [165, 176–179], with a few exceptions [164, 66]. ‘Longevity genes’, discovered via strong loss-of-function mutations in the laboratory, might thus not always harbor variants in natural populations [47, 177], even though segregating IIS polymorphisms seem to contribute to the exceptional longevity of human centenarians [147–152]. Given the conserved role of IIS/TOR in regulating life-history physiology in response to the external and internal ‘milieu’, a possible explanation for the lack of standing variation is that the plasticity of the network has been optimized by selection but that it is now under selective constraint, with most newly arising mutations being deleterious and purged by purifying selection [180, 181]. Additionally, mapping studies often work with inbred lines and homozygous effects of recessive variants that would play little role in genetic variation in life-history phenotypes in natural, outbred populations, and the experiments are conducted in a laboratory environment that is very different from that in which the fly life history has evolved.

Genomic analyses of selection experiments in flies have also revealed other mechanisms that might be important determinants of the rate of aging. Sequencing of the genomes of flies after 50 generations of longevity selection revealed a statistical enrichment of allele frequency changes at loci involved in defenses against fungal infections [178], and a similar ‘evolve and resequence’ study also identified immunity genes as candidate loci for postponed senescence [179]. The fact that over-expression of immune genes, leading to immune hyperactivity, shortens lifespan, while reduced immune signaling can promote longevity (reviewed in [182]), suggests that allele frequency changes at these loci might underlie, to some degree, evolutionary changes in the rate of aging in these experiments. Little is understood about the mechanistic interplay between immunity and aging, but it is clear from studies of both model organisms [182] and humans [183, 184] that increased inflammation (‘inflammaging’) is a major feature of the aging process, and these artificial selection experiments could provide a powerful context for analyzing the mechanisms at work.

## Is aging universal? Diverse patterns of senescence among species

**Fig. 4.**
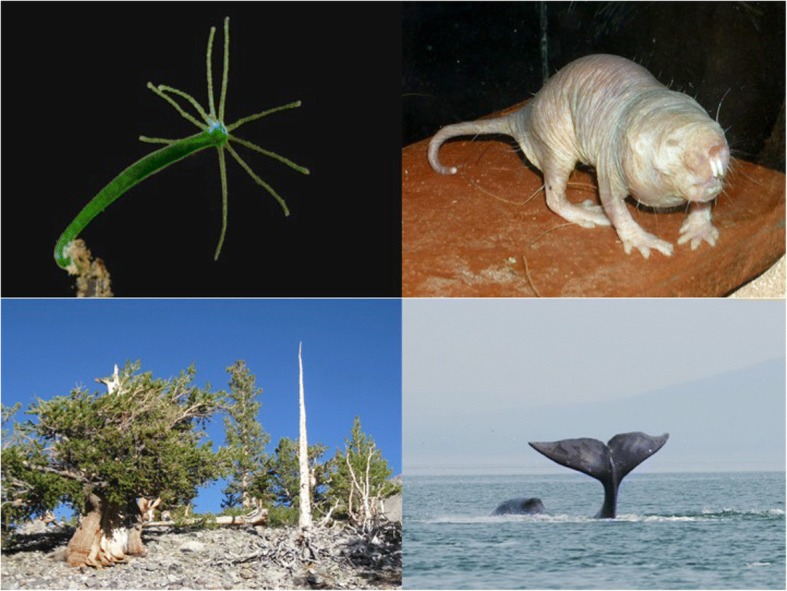
Longevous organisms. Many organisms age very slowly, if at all. Top left: the freshwater polyp *Hydra* (top left) is potentially immortal. Bottom left: some trees like this bristlecone pine (*Pinus longaeva*) live for thousands of years. Top right: in the naked mole-rat (*Heterocephalus glaber*) mortality does not increase with age. Bottom right: the bowhead whale (*Balaena mysticetus*) is the longest-lived mammal, with an estimated maximum lifespan of 211 years. Images: Hydra – © Frank Fox and www.mikro-foto.de Wikimedia Commons/CC-BY-SA-3.0; bristlecone pine - J Bre*w*/*W*ikimedia Commons/CC-SA-1.0; naked mole-rat ©Roman Klementschitz/Wikimedia Commons/CC-BY-SA-3.0/GFDL; bowhead whale - Olga Shpak/Wikimedia Commons/CC-BY-SA-3.0

Classic theories of aging pertain mainly to relatively short-lived species with increasing mortality and decreasing fertility after maturity, but patterns of aging—including reproductive senescence—are very diverse [21, 27, 185–191]. In particular, although many species do age, some appear to show ‘negligible’ senescence (i.e., only weak or no signs of aging with advancing age) [21, 27, 192–194] (Fig. 4), whereas others could—at least theoretically—exhibit ‘negative’ senescence (i.e., physiological improvement with age) [195]. In freshwater polyps of the genus *Hydra* (Fig. 4), for instance, survival and fertility do not decline with age [189]. Similarly, many plants (e.g., ~ 93% of angiosperms) show no signs of aging [196, 187]; some trees, for example, live thousands of years (Fig. 4). However, a caveat is that aging might in many cases exist but not be detectable because the studied individuals were not old enough [197]; for example, a recent study of turtles—typically thought of as exhibiting strongly ‘negligible’ senescence—has shown that reproduction and survival do in fact decline with age, contrary to previous expectations [198]. Many organisms, such as numerous invertebrates and fish, start to reproduce before they are fully grown. Increasing body size can then lead to increased fecundity and also to protection against size-specific predators and other sources of mortality. Under these circumstances, the force of natural selection can increase over part of adult life, because the reproductive value of the organism increases [25, 35, 38, 199]. Non- or slow-aging species, including some animals (e.g., basal metazoans such as *Hydra* and sea anemones) and most higher plants, are characterized by modular organization, indeterminate (including clonal) growth, and the capacity to regenerate due to stem cell activity; often such organisms start to reproduce before they have finished growing, or they can grow indefinitely [26, 200] (but see [201]). Some clones of grasses, for example, have been estimated to become 15,000 years old [202]. In addition, unlike the standard laboratory model organisms, which set aside and sequestrate their germline early in development, in organisms such as *Hydra* and higher plants the cells that will become the germline are only identified during adulthood, and these organisms therefore maintain cell lineages with high regenerative potential. Thus, the force of natural selection does not always decline monotonically with age [25–27, 35, 38].

These observations suggest that aging might not be universal [25–27], despite some claims to the contrary [203]. An obvious example of a biological system that defies senescent deterioration is the germ line itself, at least when sexual reproduction counteracts the accumulation of deleterious mutations [204–206]—indeed, already in 1885 Weismann [207] stressed that aging might be a phenomenon of the soma (also see [33]). Generally, senescence should only evolve in those organisms that have a distinction between parents and offspring, even when reproduction occurs asexually [33, 38, 44]; for example, if the parent reproduces by simple splitting or dividing symmetrically into identical offspring, then there is no clear delineation of parents versus offspring, selection cannot distinguish between them since there is no age structure, and aging is not expected to evolve [38, 44, 208]. Questions of potential immortality naturally occupy a central place in human thought: might it be possible to increase human lifespan significantly beyond the current level and in such a way that people stay healthy much longer? A recent study has claimed that such hopes are misplaced since life expectancy might reach a limit at around 115 years [14] but this analysis remains controversial [15, 16].

Organisms that have evolved extraordinary longevity are rich material for understanding how the effects of aging can be combatted, and they are usually found in nature rather than in the typical laboratory. Significant work in this direction is being carried out in several fascinating invertebrate and, importantly, vertebrate systems. In addition to the work in *Hydra* (mentioned above) and social insects (discussed below), studies of the naked mole rat (*Heterocephalus glaber*; Fig. 4), the most long-lived rodent, have revealed that it is remarkably resistant to oxidative stress and cancer [209, 210]. Intriguingly, a recent analysis based on over 3000 data points suggests that mortality rate does not increase with age in this species, even though certain physiological functions do exhibit (attenuated) senescent decline [190]. Similarly, a short-lived (median lifespan ~ 4 months) fish, the turquoise killifish (*Nothobranchius furzeri*), has been developed into a convenient organism for studying vertebrate aging [211–213]. This model is promising since these fish exhibit an array of aging traits, including cancer, can be easily reared and manipulated in the laboratory, are amenable to transgenesis and genomics, and possess natural populations that differ in their rates of aging [211]. Planarian flatworms have also recently been suggested as a promising and experimentally tractable model system, since they are potentially somatically immortal, possess pluripotent stem cells, have an amazing ability to regenerate all tissues and body parts, and are amenable to RNAi screens [211]. More work on aging and longevity is also needed in wild populations, especially in vertebrate populations (reviewed in [22, 214]). Importantly, a review of the evidence for aging in wild animals by Nussey and colleagues, based on 175 species (mainly birds and mammals but also in other vertebrates and insects) from 340 separate studies, has shown that aging is prevalent in natural populations [22].

## Trade-offs with lifespan are pervasive but can be uncoupled

Studies of natural populations have also found support for phenotypic trade-offs consistent with the notion of AP/DS [22, 197]. In bats, for example, species that produce more offspring are shorter-lived than those that produce fewer offspring [215]. Similarly, a recent review of 26 studies of free-ranging populations of 24 vertebrate species (birds, mammals, reptiles) has identified clear-cut trade-offs between early and late fitness components [53], and data in humans have unraveled a genetically based trade-off between reproduction and lifespan [78]. Trade-offs thus seem to be pervasive: high resource allocation to growth or reproduction early in life is often associated with earlier or more rapid aging. However, there is also growing evidence that trade-offs between lifespan and other fitness components are context-dependent and can be ‘uncoupled’, as is observed in some long-lived *C. elegans* or *Drosophila* mutants (reviewed in [47, 50, 166, 175]), or upon manipulation of specific dietary amino acids in flies [109, 144] (see below), without any apparent fitness costs of longevity. In these cases, a likely explanation is the artificially benign laboratory environment occupied by these organisms, which may allow them to realize their physiologically maximal possible investments into both survival and reproduction.

The most famous example of an ‘uncoupling’ of the fecundity–longevity trade-off is seen in eusocial insects (i.e., ants, bees, termites). In many ants, for example, queens are extraordinarily long-lived and highly fertile as compared to the short-lived and sterile workers [166, 216–223], even though *within* the worker caste reproductive costs have been found among fertile bumblebee workers [224]. (On the other hand, in naked mole rats, which are also eusocial, queens and workers have approximately equivalent lifespans but workers do not reproduce while queens can produce up to 900 pups [225].) How can social insect queens (or kings in termites) escape this trade-off? Surprisingly little formal analysis of this problem exists; the standard explanation that has been put forward is that queens and kings live much longer because they are shielded from extrinsic mortality by the workers [216, 219]. In addition, queens or kings may defy the fecundity–longevity trade-off because of trade-offs at the colony level [226], with resources provided by workers freeing them from individual-level trade-offs; at the colony level, queens and kings might be viewed, metaphorically, as representing the ‘immortal germline’, whereas workers can be seen as representing the ‘disposable soma’ [227]. Classic theories of aging may also not fully apply to eusocial insects [226]: their populations exhibit not only age structure but also strong social structure and division of labor. Since in such a situation survival is not only age- but also state-dependent, the force of selection does not necessarily decline with age [83]. More theoretical work on aging in eusocial insects is warranted, especially the development of class-structured inclusive fitness (kin selection) models [166, 226–228].

Beyond eusocial insects, work on Volvocalean green algae (some of which are unicellular, whereas others form multicellular colonies) suggests that division of labor might be a general principle underlying the decoupling of trade-offs [229, 230]. Unicellular algae face a trade-off between flagellar locomotion and reproduction. In these small planktonic algae, survival is dependent upon locomotory ability, i.e., staying in the water column and escaping predators. The flagellum in turn depends on the centriole, yet the centriole is also required for cell division (reproduction), so that the cell has to forgo locomotion while it divides, thereby leading to a survival-reproduction trade-off. By contrast, colonial forms of these green algae have apparently managed to uncouple the trade-off by having sterile cells devoted to motility, while other cells are specialized to perform the reproductive function—a situation akin to the differentiation of the germline and soma. A convex trade-off function, i.e., an upward-bent curve that relates survival to reproduction, should favor colony formation and specialization into separate survival (somatic) versus reproductive (germline) functions, whereas such a division of labor should not evolve when the trade-off curve is concave, i.e., bent downward [229]. This principle has been generalized, in a theoretical cost–benefit analysis of accelerating and decelerating performance functions [231].

A major aim of mechanistic research into aging is to compress morbidity at the end of life, by shortening its duration and lessening its severity. Importantly, improvement of health during aging should not be associated with adverse side effects. The pleiotropy route to the evolution of aging could be taken to imply that any amelioration of the effects of aging could be achieved only at the cost of problems earlier in adult life, because of the predicted genetic correlation between early and late fitness. However, the finding that increased lifespan can be achieved in the absence of associated costs to reproduction, both in the laboratory and in nature in the case of social insects, indicates that this correlation can be broken.

An important insight into the likely explanation for the ‘breaking’ or ‘uncoupling’ of trade-offs comes from the different outcomes of attempts to measure reproductive costs by looking at natural correlations across individuals as opposed to experimental manipulation of reproductive rate. Generally, across individuals in natural populations, there is a positive phenotypic correlation between fecundity and lifespan. However, the causal connection between the two traits may be the opposite, as experimental manipulations of, for instance, increasing clutch size in birds, often lead to reduced future fecundity or survival [232]. This difference occurs because the individual variation in condition and circumstances may obscure the underlying cost of reproduction: healthy individuals in a rich environment may have high fecundity and lifespan despite the cost of reproduction, which is only revealed by experimental manipulations. This underlying cost of reproduction may then constrain the combinations of life history traits that can evolve [233, 234]. Organisms that live in an environment that is beneficial for development may indeed not experience costs of reproduction [233, 234], as often seems to be the case in laboratory animals [235]. In addition, positive correlations between fitness-related traits can also be caused by mutational variation in recessive deleterious effects [236]. This arises because such deleterious mutations can have negative pleiotropic effects on two or more traits but the extent of these negative effects varies genetically among individuals.

Prevention of late-life morbidity in humans ideally would involve interventions that could be started at the earliest in middle age. Pharmacological prevention of cardiovascular disease, with statins and blood pressure lowerers, is already routine in clinical practice [237]. Unsurprisingly, many of the proteins that have turned out to be important in aging also play prominent roles in the etiology of age-related diseases, and are already the targets of licensed drugs. Consideration is hence starting to be given to widening the preventative, pharmacological approach, for instance by repurposing drugs such as rapamycin, which inhibits TOR and is used to treat cancer and to prevent rejection of transplanted organs, and metformin, used to treat type 2 diabetes and which may have several modes of action; importantly, both drugs have been found to extend lifespan in model organisms [238–241]. Other possible approaches to emerge from experimental work with animals include removal of damaging senescent cells that accumulate during aging [242, 243], use of factors from young blood that restore the age-related loss of function of stem cells or synapses between nerve cells in the brain [100, 244], and alteration of the composition of the microorganisms in the gut to a younger profile [245–247], which has already been shown to extend lifespan in the turquoise killifish [248].

However, despite the considerable promise of these approaches, the extent to which they can yield health benefits free of side effects needs detailed study, since they could pose some new challenges for an aged system. For instance, removal of senescent cells, or restoration of stem cell function, could be beneficial in the short term, but in the longer term could lead to stem cell exhaustion and tissue dysfunction.

## Key lessons from the evolution of aging

We conclude our brief ‘tour d’horizon’ of the evolution of aging with four key messages:

- Everything we know about the evolution of aging tells us that it is not a programmed process, so it has often been thought of as being intractable to experimental analysis or medical intervention. But evolutionarily conserved high-level regulators of phenotypic plasticity have turned out to be able to produce a major rearrangement of physiology and to ameliorate the effects of aging.
- Amelioration of aging can protect against multiple types of loss of function and age-related diseases, potentially without side effects given that we can ‘shake off’ trade-offs in some circumstances—this is, at least potentially, very good news for the compression of late-life morbidity in humans.
- Some species achieve extraordinary longevity—the longest-lived vertebrate, the Greenland shark (*Somniosus microcephalus*), reaches maturity at 150 years and lives ~ 400 years [249], and a clam, the ocean quahog (*Arcrtica islandica*), probably lives up to 500 years [250]. Notably also, bats and birds have longer lifespans than mammals with similar body sizes [251]. Although a major challenge, it will be revealing to understand a lot more about how these slow-aging creatures achieve their long lives, and whether their secrets could help to improve human health late in life.
- Some species (e.g., *Hydra*) do apparently not age. Such organisms clearly deserve much more mechanistic investigation as they might hold key lessons for regeneration and repair and thus for our understanding of how long life can be achieved.
